# Translation and adaptation of the multidimensional measure of informed choice and the decision regret scale for evaluating non-invasive prenatal test implementation in Norwegian public healthcare

**DOI:** 10.1016/j.pecinn.2026.100476

**Published:** 2026-04-05

**Authors:** Andrea Fotland Krohn-Hansen, Cathrine Bjorvatn, Jörg Assmus, Ragnhild Johanne Tveit Sekse, Ida Wiig Sørensen

**Affiliations:** a VID Specialized University, Ulriksdal 10, 5009 Bergen, Norway; b Department of Medical Genetics, Haukeland University Hospital, Jonas Lies vei 9B, 5021 Bergen, Norway; c Department of Research and Development, Haukeland University Hospital, 5021 Bergen, Norway; d Department of Global Public Health and Primary Care, University of Bergen, Årstadveien 21, 5009 Bergen, Norway

**Keywords:** Translation, Cultural adaptation, Informed decision, NIPT implementation study

## Abstract

**Objective:**

Norway recently implemented publicly funded non-invasive prenatal testing (NIPT) for pregnant women aged 35 years or older. To evaluate informed decision-making and decision regret in this context, valid and reliable Norwegian instruments are needed. This study aimed to translate, culturally adapt, and psychometrically evaluate the Multidimensional Measure of Informed Choice (MMIC) and Decision Regret Scale (DRS) for use in Norwegian antenatal care.

**Methods:**

Following established cross-cultural adaptation guidelines, we conducted forward and backward translation, expert committee review, patient representative feedback, and cognitive interviews. Psychometric evaluation included 120 pregnant women participating in the DIANA study, a Norwegian evaluation of the NIPT screening program. Participants completed the MMIC three weeks after NIPT blood sampling and the DRS four weeks later. Internal consistency (Cronbach's alpha) and content validity were assessed.

**Results:**

The Norwegian MMIC and DRS demonstrated comparable performance to the original English instruments. Adaptation identified linguistic, cultural, and medical terminology differences particularly regarding the terms «baby,” “chance,“ and “decision versus choice.” Cronbach's alpha coefficients were 0.63 for knowledge, 0.98 for attitude, and 0.82 for deliberation indicating acceptable to excellent reliability.

**Conclusions:**

This study provides the first reliable and culturally adapted Norwegian versions of the MMIC and DRS, enabling evaluation of informed decision-making and decision regret in prenatal screening.

**Innovation:**

Patient perspectives were included early in the adaptation process, and procedures followed the COSMIN study checklist standards, supporting evaluation of NIPT and other health care decisions.

## Highlights

• MMIC and DRS instruments were translated and culturally adapted for Norway.
• Cognitive interviews and expert review supported content validity.
• Instruments enable evaluation of informed choice in public NIPT.

## Graphical abstract

**Figure:**
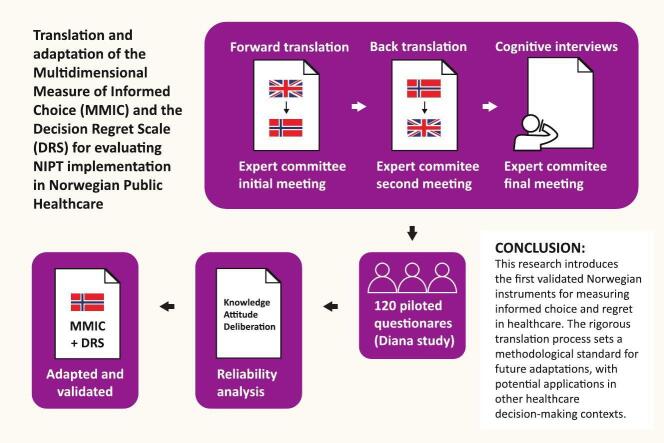

## 1 Introduction

Non-invasive prenatal testing (NIPT) has become an integral part of prenatal screening in many countries. While NIPT offers high sensitivity and specificity for common trisomies, it also presents pregnant women with complex medical and ethical decisions. Ensuring that women make informed decisions is therefore central to the ethical implementation of NIPT in public healthcare.

The DIANA study (Decisions, Information, and Autonomy in Norwegian Antenatal Care) [1] was initiated to evaluate the implementation of the non-invasive prenatal test- based screening program in Norway. The study responds to a 2021 amendment to the Norwegian Biotechnology Act, which expanded public access to trisomy screening using NIPT [2]. This amendment allows pregnant women in Norway to request trisomy screening, with public funding provided for those meeting specific criteria, such as being over age 35 at term.

Women undergoing NIPT face complex emotional and medical decisions, underscoring the need for comprehensive pre-test counselling [3], [4]. However, general practitioners, midwives, and obstetricians often lack specialized training in medical genetics and provide pre-test counselling during already busy prenatal consultations. Many health care providers are concerned that pregnant women may lack an understanding of the test's accuracy and potential for false positives, and may not be aware of their options following a high-risk result, i.e. invasive testing and/or pregnancy termination [5].

These challenges highlight the importance of assessing informed decision-making and decision regret as key indicators of the program's quality and effectiveness. Valid and reliable instruments are therefore needed to measure these outcomes. This aligns with research in other European countries, where NIPT implementation has been evaluated through studies on informed decision-making. Results indicate that women make more informed decisions about NIPT when provided with comprehensive genetic counselling and information [6], [7], [8], [9], [10].

The Multidimensional Measure of Informed Choice (MMIC) assesses whether decisions align with an individual's knowledge, values, attitudes and behaviour [11], [12], while the Decision Regret Scale (DRS) [13] evaluates post-decision distress or regret. The MMIC was initially developed for Down syndrome screening [12], and has been adapted for use in various contexts, including NIPT [6], [14], [15], [16]. Both measures are available in English; however, no validated Norwegian translations currently exist.

The purpose of this study was to translate and culturally adapt the MMIC and DRS into Norwegian, and to evaluate their psychometric properties for use in assessing informed decision-making and decision regret in the context of NIPT implementation in Norwegian public healthcare. We employed a comprehensive translation and cultural adaptation process, including content analysis and psychometric evaluation, to translate and culturally adapt the MMIC and DRS for use in the Norwegian language and healthcare setting. This article addresses the translation, cultural adaptation, and psychometric evaluation of the instruments. Analyses concerning informed decision-making, knowledge levels, and attitudes among women offered publicly funded NIPT will be presented in a separate publication from the DIANA study.

## 2 Methods

### 2.1 Study design and ethics

This was a cross-cultural translation and cultural adaptation study of MMIC and DRS, combined with a cross-sectional validation study. This study followed established guidelines for translation and cultural adaptation as specified by Wild et al. [17], Beaton et al. [18], and the COSMIN Study Design Checklist Review [19]. Content validity was assessed through expert committee review, patient representative feedback, and cognitive interviews. Psychometric evaluation was conducted using pilot data from participants in the DIANA study.

The DIANA study was reviewed by the Regional Committees for Medical and Health Research Ethics (REK) under reference number 457558. They determined it to be outside REK's jurisdiction since it is defined as a quality project. The study was registered with the Data Protection Officer at the University Hospital Eprotocol on May 22, 2023 (ID:3666). Data protection procedures were also reviewed and approved by the Data Protection Officer at Haukeland University Hospital. All participants provided informed electronic consent prior to participation. The study was conducted in accordance with the principles of the Declaration of Helsinki [20].

### 2.2 Outline of questionnaires

The MMIC comprises 52 questions across five domains: knowledge, attitude, deliberation, decisional conflict [21] and anxiety [22]. It also includes the uptake of NIPT, including whether women opt for NIPT or not. Women who have a positive attitude towards NIPT and choose the analysis are considered value-consistent [10].

The knowledge scale consists of four domains: characteristics of NIPT, how NIPT compares with standard Down syndrome screening, implications of testing, and knowledge about which conditions NIPT tests. The cutoff for good knowledge is 9 > 12, in addition to a correct answer to a single question regarding what conditions are included in the NIPT [6].

The attitude scale has five items: beneficial/harmful, important/unimportant, good thing/bad thing, desirable/undesirable, and reassuring/not reassuring. It is scored into three equal categories: positive, neutral, and negative.

Neutral attitude responders are excluded from the informed decision calculation, though results can be reported with or without this scale [6], [23].

According to van der Berg et al. [10] (2005), knowledge, deliberation and value consistency must be met to calculate an informed choice. Knowledge and deliberation are dichotomised into sufficient or insufficient knowledge and deliberation or lack of deliberation. Together with value- consistency or inconsistency leads to three dichotomous variables. The DRS is a five-item scale assessing post-decision distress or regret [13].

### 2.3 Translation and cultural adaptation

#### 2.3.1 Forward and backward translation

Two professional translators from a reputable translation agency translated MMIC and DRS from English to Norwegian. Subsequently, two additional independent translators, who were blinded to the original versions, translated the Norwegian version synthesized by the expert committee back into English.

#### 2.3.2 Expert committee review

The expert committee consisted of two genetic counsellors, one genetic counselling student, one senior consultant geneticist, one senior consultant obstetrician/gynaecologists and one specialist registrar in obstetrics/gynaecology. These professions all have responsibilities within prenatal healthcare.

The expert committee met three times. In the first meeting, they synthesized the forward translations; in the second, they refined the back-translations and finalized pre-final versions; and in the third, they incorporated pre-tester feedback. All discussions were recorded and transcribed for transparency, following the methodology outlined by Beaton et al. [18]. The final version was then prepared for piloting.

#### 2.3.3 Patient involvement

One patient representative from the national organization *1001 Days* participated in the project*.* A digital meeting was held via Microsoft Teams. During which the representative reviewed the overall DIANA study protocol, the Norwegian draft versions of the MMIC and DRS, and the planned procedure for conducting cognitive interviews with pre-testers. The discussion focused on the project's overall design and relevance from a patient perspective. The representative did not raise objections or concerns regarding the study design, questionnaires, or interview procedures. No modifications were made as a result of this consultation. The patient representative was not involved in data collection, analysis, or interpretation of results.

#### 2.3.4 Cognitive interviews with pre-testers

Pregnant women aged 35 years or older who were proficient in Norwegian served as pre-testers. These were not recruited from the DIANA cohort but were recruited separately. All pre-testers had completed first-trimester ultrasound screenings and were subsequently offered and accepted publicly funded NIPT. Recruitment was carried out by the midwife who performed the first-trimester ultrasound at the Women's Clinic at Haukeland University Hospital in Bergen, Norway.

Twenty telephone interviews were conducted with pre-testers using a standardized interview guide from the Center for Patient-Reported Data at Haukeland University Hospital [24]. Pre-testers reviewed the pre-final Norwegian versions of the MMIC and DRS prior to the interview. Each questionnaire item was examined individually, and participants were asked whether items were difficult to understand, confusing, contained unfamiliar vocabulary, were perceived as upsetting, or required rephrasing. Interviews were conducted anonymously, with no personal information collected or stored. The interviews were not audio-recorded; structured notes were taking during each session.

The main author reviewed and compiled structured notes from all interviews. Items identified by two or more pre-testers as presenting comprehension difficulties were submitted to the expert committee for further evaluation. At the third expert committee meeting, each item was systematically reviewed, and pre-tester feedback was compared with both the forward-and back-translated versions to assess the need for revisions. Modifications were implemented when wording or expressions were determined to impact comprehension, following principle outlined by Beaton et al. [18] comments reflecting individual wording or preferences that did not influence understanding were not considered sufficient for modifications.

**Fig. 1:**
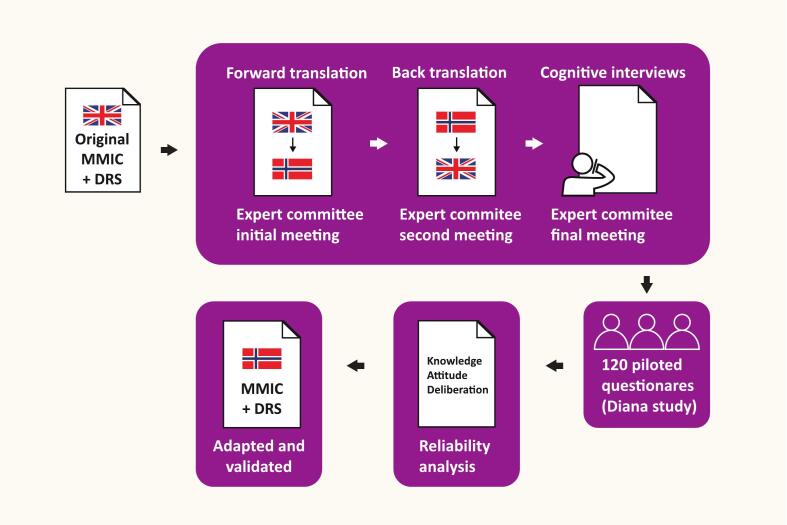
Adaptation of the MMIC and DRS.

The adaption process is shown in Fig. 1.

### 2.4 Piloting

Pilot testing was carried out with the first 120 responders in the DIANA study, as recommended [19].

From September 2023, all pregnant women aged 35 or older at term who were offered publicly funded NIPT were invited to a questionnaire-based study, regardless of their decision to undergo the test. Healthcare providers, including doctors and midwives, offered both written and oral information about the study. All participants were enrolled in our electronic study platform, where they signed the consent form and completed the questionnaires. Participants were asked to complete the MMIC questionnaire within three weeks of having blood samples drawn for NIPT, and the DRS questionnaire was to be completed four weeks later. Reminders were sent by SMS.

Demographic data were collected through the electronic platform, including variables such as age, birthplace, language, marital status, education level and previous experience with prenatal diagnosis.

### 2.5 Analysis

This study assessed the cross-cultural translation and cultural adaptation of the MMIC and DRS, followed by a cross-sectional validation consisting of content validity assessment and psychometric evaluation.

#### 2.5.1 Content validity

Content validity was evaluated through expert committee discussions, patient representative feedback, and cognitive interviews with pre-testers. The expert committee discussions focused on both forward and backward translations, conceptual clarity, cultural appropriateness, and suitability for women receiving publicly funded NIPT. Feedback from patient representatives and pre-testers was incorporated before piloting.

#### 2.5.2 Psychometric evaluation

Psychometric evaluation included descriptive statistics to characterize the pilot sample and assessment of reliability. Statistics were generated in IBM SPSS Statistics 29.0.

Reliability was evaluated using internal consistency (Cronbach's alpha), for the knowledge, attitude, and deliberation scales, to determine whether the translated scales performed similarly in Norwegian as in the original language [25].

Since the MMIC does not have a total score, Cronbach's alpha was calculated only for the ‘knowledge,’ ‘attitude,’ and ‘deliberation’ scales. The MMIC also contains other standardized questionnaires, including the Decisional Conflict Scale (DCS) and the six-item short form of the state scale of the Spielberger State-Trait Anxiety Inventory (STAI). As these have already been validated [22], [26], we choose not to include them in the validation process.

## 3 Results

### 3.1 Translation and cultural adaptation

Key discussions and solutions from the first two meetings of the expert committee are summarised in Table 1.

**Table 1:** Key discussions and solutions by the expert committee after forward and backward translations of MMIC and DRS.

Concepts	Cultural and linguistic	Medical - terminology	Discussion	Solution
Baby	x		In the Norwegian healthcare system, the term “baby” is not used, when referring to an unborn fetus. Consensus was reached unanimously.	Fetus
Chance		x	In the context of medical genetics, the term “chance” is deemed inappropriate, and is avoided by genetic counsellors and genetic	Possibility
Learning difficulties		x	Consensus was reached regarding the notable distinction between the terms “learning difficulties” and “developmental disabilities” in the context of medical genetics.	Development disabilities
Side effects	x		“Side effects” does not align well with the Norwegian language, as it is primarily a medical term. The term does not accurately describe what the participants are answering in the question, and it is difficult to relate to “side effects” in the context of NIPT. As no suitable alternative was found, it was retained.	Side effects
Decision and choice	x		The original questionnaires use “decision” and “choice” in slightly different contexts. In Norwegian, these terms are interchangeable, but the translation was kept consistent to distinguish between the two terms in the final version.	Two different Norwegian words for “decision” and “choice” were chosen.
Support to prepare for a baby with the condition	x		The term *“support”* can be translated to “*støtte*,” *“hjelp,”* or “*bistand”* in Norwegian*.* The expert committee agreed on *“hjelp*” as it is more phonetically suitable. In this context, “baby/child” is appropriate as it refers to an already-born infant.	The word that was more phonetically appropriate was chosen.

The main concepts were the terms: “baby,” “chance,” “learning difficulties,” “uptake,” “side effects,” “decision, choice,” and “support to prepare for a baby with the condition”. Each concept was categorised as either a “cultural and linguistic” issue or as an issue about “medical terminology” (which also included nuances specific to Norwegian health care). Most adaptations related to cultural and linguistic factors were minor and were resolved directly.

### 3.2 Cognitive interviews with pre-testers

Pre-testers involved 20 pregnant women who had been offered NIPT and none of the pre-testers reported difficulties understanding them. All comments were collected and discussed during the third expert committee meeting. The expert committee decided to address those questions where two or more subjects had raised an issue regarding comprehension. Comments concerning pre-testers' personal preferences – i.e. phrases they disliked or preferred to avoid – were disregarded unless they directly affected comprehension. Key discussions and solutions from the third expert committee meeting are presented in Table 2.

**Table 2:** Key discussions and solutions by the expert committee after pre-testing and cognitive interviews.

Concepts	Discussion	Solution
The informed decision test	Pre-testers provided feedback indicating that the repeated use of the word “test” throughout the questionnaire was off-putting and offensive, as it conveyed a sense of being evaluated. They expressed discomfort with the notion of being tested by the health service. To address these concerns and make the questionnaire more welcoming, the word was changed.	Replaced “test” with “questionnaire”
Invasive	The term “invasive” was challenging for some women to understand, yet no suitable alternative exists. Women offered NIPT should be informed about this term during pre-test counselling. It's a strictly medical term, not a cultural or linguistic issue.	The word “invasive” was retained
“What does a highly unlikely to be affected NIPT result mean?”	An error led to the word “unlikely” being lost in the back translation. This was discovered during the interviews as many women found the question hard to understand This prompted a discussion within the expert committee regarding the importance of this step in the translation process	“Unlikely” was put back in the sentence

### 3.3 Piloting and psychometric evaluation

The final versions of MMIC and DRS were piloted with the first 120 participants in the DIANA study. These data were used to described participant characteristics and to assess internal consistency of the translated MMIC subscales. Analysis of knowledge levels, attitudes, and informed decision-making will be reported separately in a forthcoming publication from the DIANA study. One participant was removed from the analysis as she did not meet the inclusion criteria and was registered by mistake. The question regarding previous experience with NIPT had two missing responses. Participants ‘characteristics are presented in Table 3.

**Table 3:** Characteristics of the first 120 participants in the DIANA study.

Demographic variables	Valid N	Value
Age^1^	119	36 [34, 45]
Born in Norway	119	90 (75.6%)
Native language Norwegian	119	98 (82.4%)
Marital status^2^	119	
Single		3 (2.5%)
Married		116 (97.5%)
Divorced		0
Widow		0
Living arrangement	119	
Alone		3 (2.5%)
Together with partner etc.		116 (97,5%)
Together with others		0
Highest level of education	119	
Primary		11 (0,8%)
High School		10 (8,4%)
University		108 (90,8%)
NIPT before	117	
No		93 (78,2%)
Yes		22 (18,5%)
Don't know		2 (1,7%)
Prenatal care before	119	74 (62,2%)
First trimester screening before	119	68 (57,1%)
NIPT before	119	19 (16%)
CUB (combined ultrasound blood test) before	119	11 (9,2%)

1 Median [min-max].

2 N (%), *N* = 119.

### 3.4 Reliability of MMIC subscales

Internal consistency was assessed for MMIC subscales; the Cronbach alpha coefficient was 0.63 for the knowledge scale, 0.98 for the attitude scale, and 0.82 for the deliberation scale.

## 4 Discussion and conclusion

### 4.1 Discussion

#### 4.1.1 Validity and reliability of the instruments

We aimed to generate high-quality, Norwegian-language questionnaires for evaluating recent changes in prenatal health care in Norway. The MMIC and DRS instruments, widely recognised and used in prenatal health care, can now be used to measure informed decision and regret among Norwegian-speaking women eligible for publicly funded NIPT. The adaptation process involved forward and backward translation, with contributions from an expert committee at three stages. Pre-tester feedback contributed to content validity, whereas internal consistency analysis was used to assess reliability of the questionnaire scales.

While internal consistency is generally applied to reflective models, it remains valuable for formative models like the MMIC, to assess whether individual terms and items perform consistently with the original measurement [25]. Cronbach's alpha coefficients were 0.63 for the knowledge scale, 0.98 for the attitude scale, and 0.82 for the deliberation scale, indicating strong internal consistency within the respective scales. These results align with Lewis et al. [6], developers of the MMIC for NIPT, who reported values of 0.69, 0.94 and 0.84, respectively, indicating that this tool is reliable for assessing informed decision among women offered NIPT in the Norwegian population.

#### 4.1.2 Cultural and linguistic challenges

The translation and cultural adaptation process proved crucial for ensuring that the MMIC and DRS captured the intended constructs in a Norwegian context. Our experience confirmed that even minor translation inaccuracies – such as those involving terms like ‘uptake’ and ‘side effects,’ – can affect conceptual equivalence and participant understanding. These issues likely stemmed from translators' non-medical backgrounds, underscoring the value of the expert committee and patient involvement throughout the process. The adaptation stage further highlighted cultural and linguistic nuances, where meanings of specific concepts differed from the original English versions. Consistent with findings from other translation studies [25], [27], making minor, contextually appropriate modifications allowed the questionnaires to reflect Norwegian healthcare communication better while maintaining validity.

Critical discussions within the expert committee focused mainly on the terms; “baby,” “chance,” and “decision and choice.” The expert committee was unified in recommending that the word “baby” be altered in MMIC, as Norwegian healthcare providers generally avoid this term when describing a fetus. Most midwives and physicians prefer the word “fetus” as this is seen as more neutral, clinical, and respectful. Scandinavian research supports this approach, highlighting how verbal and nonverbal cues significantly shape patient perceptions and decision-making in sensitive contexts [28].

The expert committee also pointed out that genetic counsellors and geneticists in Norway often avoid using the word “chance”(*sjanse* in Norwegian) when describing the statistical probability of a medical event because, outside of the medical field, the word often has more positive connotations—e.g., “a chance of winning the lottery” or “a chance of good weather”. The expert committee solved this by opting for the word “possibility” (*mulighet* in Norwegian), which is more neutral.

In MMIC, “choice” and “decision” have distinct meanings. In this context, a choice refers to the end product of making a decision, whereas a decision refers to the process of choosing between alternatives [10]. Similar terms with different meanings illustrate the linguistic challenges of words that may not align neatly with Norwegian usage, risking subtle shifts in meaning.

#### 4.1.3 The role of pre-testing in identifying translations errors

Other studies have noted the value of modifying the target language of questionnaires [29]. In our study, there were almost no discrepancies between feedback from pre-testers and the expert committee. Interestingly, during cognitive interviews with pre-testers, we identified a missing word in one of the questions, which the expert committee did not pick up. This significantly changed the meaning of the question. This, once again, highlights the importance of a thorough process.

### 4.2 Innovation

While we have utilized well-established, best practice methods for the translation and adaptation of MMIC and DRS, our approach innovates by exceeding conventional standards. For example, we involved patient perspectives early in the adaptation process to improve cultural relevance, a step that is often neglected by other translation and validation efforts [30]. Additionally, our piloting and reliability analysis included 119 pilot participants, surpassing COSMIN study checklist recommendations [19] and reinforcing the robustness of the adapted instruments.

In an era increasingly reliant on artificial intelligence for text translation, our study demonstrates that human expertise and input are indispensable when translating and adapting these tools. Linguistic precision and cultural fidelity are elements that automated tools cannot currently replicate, especially nuances or professional language norms. Our expert committee replaced “baby” with “fetus” to reflect clinical neutrality in Norwegian healthcare. “Chance” was substituted for “possibility,” as the former carries overly positive connotations in everyday language. These cultural adjustments illustrate the irreplaceable role of human judgement in ensuring accurate and appropriate adaptation.

The main outcome of our study is the first culturally adapted Norwegian versions of MMIC and DRS, enabling-for the first time-the evaluation of informed decision-making and decision regret in prenatal screening within Norwegian healthcare. Since no other established tools exist for this purpose, our study fills a critical gap. The DIANA study itself-assessing a new, nationwide NIPT-based trisomy screening program-represents significant innovation within the field of service evaluation and patient autonomy monitoring.

Beyond the specific context, these instruments have scalable potential. They can be adapted for use across various healthcare settings where informed decisions and emotional outcomes are essential, setting precedent for culturally grounded instruments adaptation in non-English speaking health systems.

#### 4.2.1 Strengths and limitations

The reliability analysis revealed that some scales, such as the knowledge scale, achieved only moderate internal consistency which may limit the robustness of specific constructs.

Translation challenges, including the absence of direct Norwegian equivalents for some terms, highlight the risk of subtle shifts in meaning and cultural misalignment, despite rigorous adaptation procedures. While expert committee work ensured linguistic precision and cultural relevance, the process could have been further strengthened by including the original developer, as recommended by the COMSIN checklist. Their input might have helped resolve wording compromises that arose due to lack of a preferred alternative.

A methodological strength of this study was that most members of the expert committee were proficient in both English and Norwegian. This facilitated direct comparison between the original and translated versions and enabled detailed discussions of linguistic nuances and cultural adaptation. However, as in all cross-cultural translation processes, final wording decisions were based on expert judgement, and some degree of subjectivity cannot be entirely excluded.

Although professional translators from a reputable agency were employed, engaging translators with specialized health knowledge can enhance concept equivalence and reduce the need for interpretative compromises. Similarly, the expert committee could have been strengthened by including a linguist to ensure sematic precision and by involving a representative from the pilot testers to increase contextual alignment. Finally, despite the pilot sample size exceeding methodological recommendations, it may still not fully represent the diversity of the Norwegian population, thereby limiting the generalizability of the findings.

### 4.3 Conclusion

This study has successfully translated and culturally adapted the MMIC and the DRS for use in Norway, ensuring their reliability in assessing informed decision-making and regret related to NIPT. The scales for knowledge, attitude, and deliberation in the MMIC showed acceptable internal consistency, supporting their validity in this new context. This research introduces the first Norwegian instruments for measuring informed decisions and regret in prenatal care. The rigorous translation process establishes a methodological standard for future adaptations and has potential applications in other healthcare decision-making contexts.

## CRediT authorship contribution statement

**Andrea Fotland Krohn-Hansen:** Writing – original draft. **Cathrine Bjorvatn:** Writing – review & editing, Supervision. **Jörg Assmus:** Formal analysis. **Ragnhild Johanne Tveit Sekse:** Writing – review & editing, Supervision. **Ida Wiig Sørensen:** Writing – review & editing, Supervision.

## Declaration of competing interest

The authors declare that they have no known competing financial interests or personal relationships that could have appeared to influence the work reported in this paper.

## Appendix A Supplementary data

Supplementary material 1 Image 1

Supplementary material 2 Image 2
