# Supplementary material for: Translation and adaptation of the multidimensional measure of informed choice and the decision regret scale for evaluating non-invasive prenatal test implementation in Norwegian public healthcare
Source: PEC Innov. 2026 Apr 5;8:100476. doi: 10.1016/j.pecinn.2026.100476 (PMC13091119; doi:10.1016/j.pecinn.2026.100476)
Supplement: Supplementary file 1 — Supplementary material 1 [file mmc1.docx]

**(*Decision Regret Scale*)**

**Skala for anger over avgjørelsen**

Tenk over avgjørelsen du tok angående NIPT etter å ha snakket med

lege/jordmor. Sett en ring rund et tall mellom 1 (helt enig) til 5 (helt uenig) for å vise hva du synes om påstandene.

|  | Helt enig | Enig | | Verken enig eller uenig | | Uenig | | Helt uenig | |
| --- | --- | --- | --- | --- | --- | --- | --- | --- | --- |
| 1. Det var den riktige avgjørelsen | 1 | 2 | 3 | | 4 | | 5 | |  |
| 2. Jeg angrer på valget mitt | 1 | 2 | 3 | | 4 | | 5 | |  |
| 3. Jeg ville tatt samme valg igjen | 1 | 2 | 3 | | 4 | | 5 | |  |
| 4. Valget var svært skadelig for meg | 1 | 2 | 3 | | 4 | | 5 | |  |
| 5. Det var en klok avgjørelse | 1 | 2 | 3 | | 4 | | 5 | |  |
