# Supplementary material for: Translation and adaptation of the multidimensional measure of informed choice and the decision regret scale for evaluating non-invasive prenatal test implementation in Norwegian public healthcare
Source: PEC Innov. 2026 Apr 5;8:100476. doi: 10.1016/j.pecinn.2026.100476 (PMC13091119; doi:10.1016/j.pecinn.2026.100476)
Supplement: Supplementary file 2 — Supplementary material 2 [file mmc2.docx]

**SKJEMA1(Multidimensional Measure of Informed choice)**

| 1. **Svar på følgende spørsmål ett for ett. Ikke les videre til slutten av denne delen før du begynner, da det kan avsløre noen av svarene og ikke gjenspeile din virkelige forståelse av testen.**   ***NB! Det er viktig at du Ikke går tilbake for å endre på svar etter at du har gjort deg ferdig med spørreskjemaet.*** |
| --- |

**1. Hvilke(n) av disse tilstandene kan avdekkes hos fosteret med ikke-invasiv prenatal test (NIPT)? (Velg kun ett svar.)**

| Ryggmargsbrokk |  |
| --- | --- |
|  |  |
| Anemi |  |
|  |  |
| Trisomi 21 (Down syndrom) |  |
|  |  |
| Trisomi 21 (og de to sjeldnere kromosomavvikene trisomi 13 og 18) |  |
|  |  |
| Alle kjente genetiske lidelser |  |
|  |  |
| Usikker |  |

**2. Hvordan utføres NIPT? (Velg kun ett svar.)**

| Spyttprøve fra mor |  |
| --- | --- |
|  |  |
| Urinprøve fra mor |  |
|  |  |
| Blodprøve fra mor |  |
|  |  |
| Invasiv test der det tas prøve av fostervannet |  |
|  |  |
| Usikker |  |

**3. Hva betyr det hvis resultatet av NIPT viser «høy sannsynlighet»? (Velg kun ett svar.)**

| Det er helt sikkert at fosteret har denne tilstanden |  |
| --- | --- |
|  |  |
| Det er svært sannsynlig at fosteret har denne tilstanden, men det er nødvendig med invasiv testing for å bekrefte diagnosen |  |
|  |  |
| Usikker |  |

**4. Hva betyr det hvis resultatet av NIPT viser «lav sannsynlighet»? (Velg kun ett svar.)**

| Det er helt sikkert at fosteret ikke har denne tilstanden |  |
| --- | --- |
|  |  |
| Det er ikke sannsynlig at fosteret har denne tilstanden, men siden testen ikke er 100% nøyaktig, finnes det en liten mulighet for at resultatet er feil |  |
|  |  |
| Usikker |  |

**5. Hvordan er NIPT sammenlignet med tidligere screeningtest, KUB (kombinert ultralydundersøkelse og blodprøve fra mor)? (Velg kun ett svar.)**

| Den er mindre nøyaktig |  |
| --- | --- |
|  |  |
| Den er like nøyaktig |  |
|  |  |
| Den er mer nøyaktig |  |
|  |  |
| Usikker |  |

**6. Hvor trygg er NIPT? (Velg kun ett svar.)**

| Det er ingen risiko for deg eller barnet |  |
| --- | --- |
|  |  |
| Det er risiko for spontanabort |  |
|  |  |
| Usikker |  |
|  |  |

**7. Hvor lang tid tar det å få NIPT-resultatet? (Velg kun ett svar.)**

| Resultatet blir tilgjengelig umiddelbart etter at blodprøven er tatt |  |
| --- | --- |
|  |  |
| Det tar 24 timer å få resultatet |  |
|  |  |
| Det tar 7-10 virkedager å få resultatet |  |
|  |  |
| Usikker |  |

**8. Får du alltid et testresultat? (Velg kun ett svar.)**

| Ja, du får helt sikkert et testresultat |  |
| --- | --- |
|  |  |
| Nei, i noen få tilfeller kan ikke laboratoriet gi noe resultat, og testen kan gjentas |  |
|  |  |
| Usikker |  |
|  |  |

**9. Hvor trygge er invasive tester (fostervannsprøve eller morkakeprøve)?** **(Velg kun ett svar.)**

| Det er ingen risiko for deg eller fosteret |  |
| --- | --- |
|  |  |
| Det er en liten (rundt 1 %) risiko for spontanabort |  |
|  |  |
| Det er høy (20 %) risiko for spontanabort |  |
|  |  |
| Ingen av disse |  |
|  |  |
| Usikker |  |

**10. Hvis det blir bekreftet at fosteret helt sikkert har denne tilstanden, hvilke tilbud har du?
(Velg alle riktige svar.)**

| Umiddelbar behandling av fosteret |  |
| --- | --- |
|  |  |
| Hjelp til å forberede deg på å få et barn med tilstanden |  |
|  |  |
| Mulighet til å avslutte svangerskapet, hvis det er det du ønsker |  |
|  |  |
| Ingen av disse |  |
|  |  |
| Usikker |  |

**11. Er du nødt til å ta noen av disse testene? (Velg kun ett svar.)**

| Ja, alle kvinner må ta disse testene i løpet av svangerskapet |  |
| --- | --- |
|  |  |
| Nei, det er mitt eget valg om jeg vil ta disse testene eller ikke |  |
|  |  |
| Usikker |  |
|  |  |

**12. Hva er Trisomi 21 (Down syndrom)? (Velg kun ett svar.)**

| En livsvarig tilstand som gir utviklingshemning |  |
| --- | --- |
|  |  |
| En tilstand som kan kureres med kirurgi |  |
|  |  |
| En tilstand som barn vokser av seg |  |
|  |  |
| Usikker |  |

| **B. Sett en ring rundt det tallet mellom 0 og 4 som best beskriver hvordan du har det akkurat nå.** |
| --- |

**13.** **For meg ville det å ta NIPT være:**

| Gunstig | 0 | 1 | 2 | 3 | 4 |  | Skadelig |
| --- | --- | --- | --- | --- | --- | --- | --- |

**14.** **For meg ville det å ta NIPT være:**

| Viktig | 0 | 1 | 2 | 3 | 4 |  | Uviktig |
| --- | --- | --- | --- | --- | --- | --- | --- |

**15****. For meg ville det å ta NIPT være:**

| Bra | 0 | 1 | 2 | 3 | 4 |  | Dårlig |
| --- | --- | --- | --- | --- | --- | --- | --- |

**16.** **For meg ville det å ta NIPT være:**

| Betryggende | 0 | 1 | 2 | 3 | 4 |  | Ikke betryggende |
| --- | --- | --- | --- | --- | --- | --- | --- |

**17. For meg ville det å ta NIPT være**

| Ønskelig | 0 | 1 | 2 | 3 | 4 |  | Ikke ønskelig |  |
| --- | --- | --- | --- | --- | --- | --- | --- | --- |
| **C. Oppslutning** | | | | | | | | |

**18. Valgte du å ta NIPT? (Velg kun ett svar.)**

| Ja, jeg valgte å ta NIPT |  |
| --- | --- |
|  |  |
| Nei, jeg valgte å ikke ta NIPT |  |
|  |  |
| Jeg har ikke bestemt meg |  |
|  |  |

| **D. Reflekter over avgjørelsen du tok om å ta NIPT eller ikke ta NIPT. Sett en ring rundt tallet mellom 0 (helt enig) og 4 (helt uenig) som stemmer best med ditt syn på avgjørelsen din.** |
| --- |

**19. Jeg har prøvd å vurdere alternativene**

| Helt enig | 0 | 1 | 2 | 3 | 4 | Helt uenig |
| --- | --- | --- | --- | --- | --- | --- |

**20. Jeg har forestilt meg hvordan jeg ville følt meg hvis jeg ikke hadde tatt NIPT**

| Helt enig | 0 | 1 | 2 | 3 | 4 | Helt uenig |
| --- | --- | --- | --- | --- | --- | --- |

**21. Jeg har forestilt meg hvordan jeg ville følt meg hvis jeg** **hadde tatt NIPT**

| Helt enig | 0 | 1 | 2 | 3 | 4 | Helt uenig |
| --- | --- | --- | --- | --- | --- | --- |

**22. Jeg har prøvd å tenke over konsekvensene av å takke nei til NIPT**

| Helt enig | 0 | 1 | 2 | 3 | 4 | Helt uenig |
| --- | --- | --- | --- | --- | --- | --- |

**23. Jeg har prøvd å tenke over konsekvensene av å takke ja til NIPT**

| Helt enig | 0 | 1 | 2 | 3 | 4 | Helt uenig |
| --- | --- | --- | --- | --- | --- | --- |

**24. Jeg har laget en liste (i hodet) over fordeler og ulemper ved NIPT**

| Helt enig | 0 | 1 | 2 | 3 | 4 | Helt uenig |
| --- | --- | --- | --- | --- | --- | --- |

| **E. Tenk nå på de ulike alternativene du fikk for fosterdiagnostikk: NIPT, fostervannsprøve eller ingen fosterdiagnostikk. Svar på følgende spørsmål på grunnlag av det du valgte.** |
| --- |

**25. Jeg vet hvilke alternativer som er tilgjengelig for meg.**

| Helt enig | 0 | 1 | 2 | 3 | 4 | Helt uenig |
| --- | --- | --- | --- | --- | --- | --- |

**26. Jeg kjenner fordelene ved hvert av alternativene**

| Helt enig | 0 | 1 | 2 | 3 | 4 | Helt uenig |
| --- | --- | --- | --- | --- | --- | --- |

**27. Jeg kjenner risikoene og bivirkningene ved hvert av alternativene**

| Helt enig | 0 | 1 | 2 | 3 | 4 | Helt uenig |
| --- | --- | --- | --- | --- | --- | --- |

**28. Det er klart for meg hvilke fordeler som er viktigst for meg**

| Helt enig | 0 | 1 | 2 | 3 | 4 | Helt uenig |
| --- | --- | --- | --- | --- | --- | --- |

**29. Det er klart for meg hvilke risikoer og bivirkninger som er viktigst for meg**

| Helt enig | 0 | 1 | 2 | 3 | 4 | Helt uenig |
| --- | --- | --- | --- | --- | --- | --- |

**30. Det er klart for meg om det er fordelene eller de potensielle risikoene som er viktigst for meg**

| Helt enig | 0 | 1 | 2 | 3 | 4 | Helt uenig |
| --- | --- | --- | --- | --- | --- | --- |

**31. Jeg har nok støtte fra andre til å ta et valg**

| Helt enig | 0 | 1 | 2 | 3 | 4 | Helt uenig |
| --- | --- | --- | --- | --- | --- | --- |

**32. Jeg velger uten press fra andre**

| Helt enig | 0 | 1 | 2 | 3 | 4 | Helt uenig |
| --- | --- | --- | --- | --- | --- | --- |

**33. Jeg har fått nok råd til å kunne gjøre et valg**

| Helt enig | 0 | 1 | 2 | 3 | 4 | Helt uenig |
| --- | --- | --- | --- | --- | --- | --- |

**34. Det er klart for meg hva som er det beste valget for meg**

| Helt enig | 0 | 1 | 2 | 3 | 4 | Helt uenig |
| --- | --- | --- | --- | --- | --- | --- |

**35. Jeg føler meg sikker på valget mitt**

| Helt enig | 0 | 1 | 2 | 3 | 4 | Helt uenig |
| --- | --- | --- | --- | --- | --- | --- |

**36. Det er en lett avgjørelse for meg å ta**

| Helt enig | 0 | 1 | 2 | 3 | 4 | Helt uenig |
| --- | --- | --- | --- | --- | --- | --- |

**37. Jeg føler at jeg har tatt et informert valg**

| Helt enig | 0 | 1 | 2 | 3 | 4 | Helt uenig |
| --- | --- | --- | --- | --- | --- | --- |

**38. Min avgjørelse viser hva som er viktig for meg**

| Helt enig | 0 | 1 | 2 | 3 | 4 | Helt uenig |
| --- | --- | --- | --- | --- | --- | --- |

**39. Jeg forventer å holde fast ved min avgjørelse**

| Helt enig | 0 | 1 | 2 | 3 | 4 | Helt uenig |
| --- | --- | --- | --- | --- | --- | --- |

**40. Jeg er fornøyd med min avgjørelse**

| Helt enig | 0 | 1 | 2 | 3 | 4 | Helt uenig |
| --- | --- | --- | --- | --- | --- | --- |

| **F. Nedenfor finner du noen utsagn som folk har brukt for å beskrive seg selv. Les hvert utsagn og sett en ring rundt det tallet som best beskriver dine følelser rundt din graviditet nå. Det finnes ingen riktige og gale svar. Ikke bruk for mye tid på hvert utsagn, men svar det som du synes best beskriver hvordan du føler deg akkurat nå.** |
| --- |

|  | Ikke i det hele tatt | Litt | Noe | Veldig |
| --- | --- | --- | --- | --- |
| **41. Jeg føler meg rolig** | 1 | 2 | 3 | 4 |
| **42. Jeg er anspent** | 1 | 2 | 3 | 4 |
| **43. Jeg er opprørt** | 1 | 2 | 3 | 4 |
| **44. Jeg er avslappet** | 1 | 2 | 3 | 4 |
| **45. Jeg føler meg tilfreds** | 1 | 2 | 3 | 4 |
| **46. Jeg er bekymret** | 1 | 2 | 3 | 4 |
|  |  |  |  |  |

**G. Grunner til at du takket ja eller nei til NIPT**

**47. Snakket/drøftet du med jordmor/lege (på KK) om NIPT samme dag som NIPT-blodprøven ble tilbudt eller en annen dag? *(Velg kun ett svar.)***

| Testen var tilgjengelig samme dag |  |
| --- | --- |
|  |  |
| Testen var tilgjengelig en annen dag |  |
|  |  |
| Testen var tilgjengelig samme dag, men jeg valgte å komme tilbake en annen dag |  |
|  |  |

**Hvis du takket ja til NIPT, gå til spørsmål 48. Hvis du takket nei til NIPT, gå til spørsmål 50.**

**48. Hvilke(t) utsagn samsvarer mest med dine grunner til å takke ja til NIPT? *(Velg opptil to svar.)***

| Slik at jeg kan planlegge for, og forberede meg på, å føde et barn med trisomi 21 (Down syndrom) | |  | |
| --- | --- | --- | --- |
|  | |  | |
| For å hjelpe meg med å avgjøre om jeg skal fortsette svangerskapet eller ikke | |  | |
|  | |  | |
| For å forsikre meg om at fosteret ikke har trisomi 21 | |  | |
|  | |  | |
| For å unngå å få et barn med trisomi 21 | |  | |
|  | |  | |
| Fordi det ikke er noen risiko for fosteret | |  | |
|  | |  | |
| Fordi partneren eller familien min ønsker det | |  | |
|  | |  | |
| Fordi jeg fikk tilbudet som en del av svangerskapsomsorgen | |  | |
| Annet: __________________________________________________________________ |  | |  |

**49. Hvilken enkeltfaktor var viktigst for din avgjørelse om å takke ja til NIPT? *(Velg kun ett svar.)***

| Fosterets sikkerhet (ingen risiko for spontanabort) |  |
| --- | --- |
|  |  |
| Resultatet er tilgjengelig tidlig i svangerskapet |  |
|  |  |
| Nøyaktige resultater |  |
|  |  |
| At testen er lett tilgjengelig |  |
|  |  |
| At testen er enkel å ta |  |

**Hvis du takket nei til NIPT:**

**50. Hvilke(t) utsagn) samsvarer mest med dine grunner til å takke nei til NIPT? *(Velg opptil to svar.******)***

| Jeg ville aldri avsluttet svangerskapet hvis fosteret har trisomi, så det ville ikke vært noe poeng i å ta testen | |  | |
| --- | --- | --- | --- |
|  | |  | |
| Det ville skape mye engstelse hvis fosteret hadde trisomi | |  | |
|  | |  | |
| Jeg ønsket ikke å måtte ta en avgjørelse om hvorvidt å avbryte svangerskapet | |  | |
|  | |  | |
| Partneren eller familien min ville ikke ønsket at jeg tok testen | |  | |
|  | |  | |
| Jeg foretrekker å ikke vite | |  | |
| Annet: ___________________________________________________________________ |  | |  |
